# Administration of Multivalent Influenza Virus Recombinant Hemagglutinin Vaccine in Combination-Adjuvant Elicits Broad Reactivity Beyond the Vaccine Components

**DOI:** 10.3389/fimmu.2021.692151

**Published:** 2021-07-14

**Authors:** Jenny E. Hernandez-Davies, Jiin Felgner, Shirin Strohmeier, Egest James Pone, Aarti Jain, Sharon Jan, Rie Nakajima, Algimantas Jasinskas, Erwin Strahsburger, Florian Krammer, Philip L. Felgner, D. Huw Davies

**Affiliations:** ^1^Vaccine Research and Development Center, Department of Physiology and Biophysics, School of Medicine, University of California, Irvine, Irvine, CA, United States; ^2^Department of Microbiology, Icahn School of Medicine at Mount Sinai, New York, NY, United States

**Keywords:** vaccine, influenza, adjuvant, CpG, MPLA, ADDAVAX^®^, hemagglutinin

## Abstract

Combining variant antigens into a multivalent vaccine is a traditional approach used to provide broad coverage against antigenically variable pathogens, such as polio, human papilloma and influenza viruses. However, strategies for increasing the breadth of antibody coverage beyond the vaccine are not well understood, but may provide more anticipatory protection. Influenza virus hemagglutinin (HA) is a prototypic variant antigen. Vaccines that induce HA-specific neutralizing antibodies lose efficacy as amino acid substitutions accumulate in neutralizing epitopes during influenza virus evolution. Here we studied the effect of a potent combination adjuvant (CpG/MPLA/squalene-in-water emulsion) on the breadth and maturation of the antibody response to a representative variant of HA subtypes H1, H5 and H7. Using HA protein microarrays and antigen-specific B cell labelling, we show when administered individually, each HA elicits a cross-reactive antibody profile for multiple variants within the same subtype and other closely-related subtypes (homosubtypic and heterosubtypic cross-reactivity, respectively). Despite a capacity for each subtype to induce heterosubtypic cross-reactivity, broader coverage was elicited by simply combining the subtypes into a multivalent vaccine. Importantly, multiplexing did not compromise antibody avidity or affinity maturation to the individual HA constituents. The use of adjuvants to increase the breadth of antibody coverage beyond the vaccine antigens may help future-proof vaccines against newly-emerging variants.

## Introduction

Vaccines save millions of lives annually and represent one of the most cost-effective medical interventions for controlling infectious disease and the threat from pandemics. Many once commonplace childhood diseases have been eliminated through concerted deployment of vaccines. Vaccination will become even more important in the face of rising antibiotic resistance and as the number of new antibiotics in the developmental pipeline dwindles (1, 2). Despite the success of vaccination, achieving protection against rapidly evolving pathogens remains a challenge. This may be overcome by a vaccine that elicits cross-reactivity between variants, or by a multivalent approach that elicits multiple specific responses.

Influenza virus is a model human pathogen that has led to breakthroughs in understanding many key immunological principles (3–5), and has been an ideal system for the development of several novel technologies, including nucleic acid vaccination (6), human mAb production (7) and modeling the adaptive immune response *ex vivo* (8). Globally, seasonal influenza results in 290,000-650,000 million deaths annually from respiratory disease (9). Seasonal influenza is typically caused by type A and B influenza viruses. These are RNA viruses that possess an RNA-dependent RNA polymerase that is error-prone and lacks proof-reading function. Consequently, influenza viruses acquire mutations as they replicate. Influenza A viruses have a broad host range that includes birds, such as poultry and waterfowl, and mammals, such as swine and humans. Influenza B viruses do not exhibit the same strain diversity as type A and are found mainly in human hosts, and rarely, as a reverse zoonosis in seals (10).

The major target of influenza virus neutralizing antibodies is the immunodominant hemagglutinin (HA) on the virion surface, a sialic acid receptor that facilitates viral attachment to host respiratory epithelial cells. Eighteen different phylogenetically distinct subtypes of HA have emerged in influenza A viruses (H1 to H18). Acquisition of amino acid substitutions in neutralizing epitopes in the HA can lead to escape of antibody recognition and such ‘escape mutant’ viruses thrive under selective pressure from the immune system. The minor target of the neutralizing antibody response is the immune-subdominant neuraminidase (NA) on the virion surface. NA is an enzyme that facilitates viral egress from the host cells, and also exists in phylogenetic subtypes (N1-N9). Although many combinations of HA and NA are possible, only two (H1N1 and H3N2) are currently circulating in human populations. Two influenza-like genomes (H17N10 and H18N11) were recently described in bats, although neither the HA or NA proteins have the same functions found in influenza viruses (11, 12).

Evolution of HA under immune selective pressure allow the viruses to escape antibody recognition, a process known as “antigenic drift”, leading to loss of vaccine protection and new seasonal outbreaks. This causes the seasonal influenza vaccine to lose efficacy over time and requires that the variants used to formulate the vaccine to be adjusted for each new season. Current seasonal influenza vaccines comprise a tri- or quadravalent cocktail of inactivated H1N1 and H3N2 viruses, plus an additional one or two type B strains, of either the Yamagata or Victoria lineages, to provide wider coverage. To reduce reactogenicity, the membrane fraction is isolated from other virion components (so-called “split” vaccine) and administered without adjuvant, although the lack of adjuvant comes at the cost of reduced immunogenicity and durability of the response (13, 14). Protection is also confined to the strains included in the vaccine, which is quickly rendered ineffective by antigenic drift (15). As a consequence, the strains represented of seasonal vaccines are adjusted and re-administered annually. There is thus an urgent need for improved influenza vaccines with broader and longer-lived protection.

Improved influenza vaccines are needed to elicit a broader response to cover newly emerging strains. Ideally this should comprise cross-reactive antibodies (and T cells) to regions that are conserved between variants, such as receptor binding-sites. This approach is desirable since it could provide anticipatory protection against the emergence of future variants. Current strategies towards achieving this aim include ways to boost the response to conserved regions, such as sequential immunization of hemagglutinin (HA) variants (16, 17), immunizations with “headless” HA (18, 19) to drive the response to the conserved stem domain, or immunization with conserved epitopes (20). However, viral vulnerabilities are usually hidden from antibody recognition by conformational masking (21) or by shrouding by glycans (22, 23). Similar strategies are also employed by HIV (24, 25). In influenza virus, such regions are typically subdominant to the more variable epitopes in the head of the HA protein that dominate the focus of the neutralizing antibody response (26–30). A parallel approach to achieving broad protection is the multivalent vaccine approach, in which a cocktail of variant antigen strains is administered. In this way, broad reactivity is achieved through the combined sum of individual variant-specific responses. A potential disadvantage is that it is less anticipatory than approaches that intentionally target conserved regions, and therefore may be easily evaded by a rapidly evolving pathogen. Also, for reasons that are not well understood, combining variant antigens is known to sometimes thwart affinity maturation to individual constituents (31).

Subunit vaccines based on recombinant proteins may help overcome many of the shortcomings of conventional vaccine approaches, but require adjuvants to engender a robust immune response and memory (32–34). In particular, unmethylated oligodeoxynucleotides (ODN) with cytosine-guanine (CpG) motifs are recognized by toll-like receptor-9 (TLR9) on antigen presenting cells (APCs) (35–37). Similarly, monophosphoryl lipid A (MPLA), the TLR4-binding component of bacterial lipopolysaccharide (LPS), retains the immunostimulatory properties of LPS without reactogenicity (38, 39). Both CpG and MPLA are next generation adjuvants used in a number of recently-approved vaccines (40–42). Although used individually, there is increasing awareness that combining adjuvant components increases the efficacy of vaccines (43–49).

In this study, we have used a combination adjuvant comprising MPLA, CpG ODN1018, and AddaVax™ (a squalene-in-water emulsion) to formulate recombinant trimers of influenza virus hemagglutinins H1, H5 or H7 into monovalent or trivalent vaccines, and evaluated the breadth of the antibody response. We found each HA subtype was able to engender broad homosubtypic cross-reactivity, as well as significant heterosubtypic cross-reactivity for closely-related subtypes. Combining the antigens as a trivalent cocktail produced a profile that was far broader than the profiles elicited by any one of the individual subtypes. Moreover, combining the antigens appeared to have no detrimental effect on the breadth of the response, and did not appear to compromise the maturation of the response to each individual component. Thus, combining HA subtypes leads to a broader antibody cross-reactivity profile than can be achieved using a single subtype, at least when full-length HA is used.

## Materials and Methods

### Antigens

Trimerized hemagglutinins (HAs) H1 from A/California/07/2009 (Cal09), H5 from A/Vietnam/1203/04 (VN04), and H7 A/Anhui/1/2013 (AH13) were expressed in insect cells and purified *via* affinity chromatography using Ni-NTA-resin (50). Each protein has a thrombin cleavage site followed by a 29 aa residue T4 ‘foldon’ trimerization motif and a 6x polyhistidine tag engineered in place of the transmembrane and endodomains. Monomeric HA (full-length HA0 and HA1 regions) were purchased from SinoBiological Inc. (Beijing, China).

### Viruses

Reassortant influenza virus A/California/07/2009 (H1N1) x A/Puerto Rico/8/1934 was obtained from BEI Resources (Manassas, VA; catalog #NR-44004), and A/Vietnam/1194/2004 (H5N1) x A/Puerto Rico/8/1934 and A/Shanghai/2/2013 x A/Puerto Rico/8/1934 were obtained from the National Institute for Biological Standards & Controls (NIBSC, South Mimms, UK; catalog #s NIBRG-14 and NIBRG-267, respectively). Each were supplied as amniotic fluids from inoculated hen eggs. H7 from A/Shanghai/2/2013 and A/Anhui/1/2013 differ by 2 aa (99.64%); H5 from A/Vietnam/1194/2004 and A/Vietnam/1203/2004 differ by 1 aa (99.82%). Viruses were propagated in Madin-Darby Canine Kidney (MDCK) cells obtained from ATCC (Manassas, VA; catalog #CCL334) according to published methods (51). Briefly, cells seeded into 150 cm^2^ flasks were incubated for 16–24 h at 37°C/5% CO_2_ in a humidified atmosphere. For inoculation, actively growing MDCK cells (80-90% confluent) were washed twice with PBS and 2ml of inoculation culture added for 1h with periodic agitation. Inoculation cultures were prepared from amniotic fluid stocks diluted to 1:50 and 1:100 in EMEM-BSA-TPCK medium, comprising Eagle’s Minimum Essential Medium (EMEM; ATCC catalog #30-2003), 0.6% bovine serum albumin (Sigma A8412) and 1μg/ml L-(tosylamido-2-phenyl) ethyl chloromethyl ketone (TPCK)-treated trypsin (Worthington Biochemical Corp; catalog #TRTPCK). After washing in PBS, infected cells were cultured in EMEM-BSA-TPCK medium supplemented with 0.2% fetal bovine serum (ATCC 30-2020) for 48-72 hours. When >90% of cells showed cytopathic effect, culture supernatants were harvested and clarified by centrifugation (3,200xg at 4°C for 10 min), and 0.5-1ml aliquots stored in cryovials at −80°C. Virus titers were determined using hemagglutination of turkey erythrocytes (Rockland Immunochemicals, Inc. Limerick, PA) as described (51). Hemagglutination units (HAU) were read as the reciprocal of the last dilution that gives rise to hemagglutination.

### Liposomes

Synthetic 1,2-distearoyl-sn-glycero-3-phosphoethanolamine-N-[dibenzocyclooctyl(polyethylene glycol)-2000 (DBCO-DSPE-PEG2000), 1,2-dioleoyl-sn-glycero-3-phosphocholine (DOPC), 1,2-dioleoyl-sn-glycero-3-phospho-(1’-rac-glycerol) (DOPG), 1,2-dipalmitoyl-sn-glycero-3-phosphocholine (DPPC), 1,2-dipalmitoyl-sn-glycero-3-phosphoethanolamine-N-[methoxy(polyethylene glycol)-750] (DPPE-PEG750), 1,2-dioleoyl-sn-glycero-3-phosphoethanolamine-N-(lissamine rhodamine B sulfonyl) (Rhod PE) were purchased from Avanti Polar Lipids Inc. (Alabaster, AL). Cholesterol (CHOL), sucrose, HEPES, phosphate buffer saline was purchased from Sigma (St. Louis, MO). Unsaturated phospholipid liposomes (so-called “fluid liposomes”) were manufactured by combining DOPC, DOPG, DBCO-DSPE-PEG2000, and RhodPE 20 μmole total lipid at the molar ratio 92/5/2.5/0.5 in chloroform organic solvent. The organic solvent was then evaporated under nitrogen, and the residual solvent was removed overnight under vacuum. The lipid film was hydrated with 1 ml of 10% sucrose/20mM HEPES buffer (pH 7.3) and sonicated in a sonicating water bath (Branson M1800) at room temperature (20-25 °C) for 15 min or until the formulation was translucent with no large visible particles. The same procedure was followed for saturated phospholipid liposome (DPPC/DPPE-PEG750/CHOL/DBCO-DSPE-PEG2000/Rhod PE at molar ratio of 63/4/30/2.5/0.5) (so called “solid liposomes”) except sonication was performed at 55-60°C. Particle size distribution of fluid and solid liposomes was found to be 100 nm and 170 nm, respectively, by dynamic light scattering (DLS) using a Malvern Zetasizer.

### Conjugation of HA Trimers to Liposomes

HA proteins were conjugated *via* the hexahistidine tags to DBCO-derivatized phospholipids on the liposomal outer layer *via* Tris-NiNTA-azide reagents (a generous gift of Tyler Albin and Aaron Esser-Khan, UCI Department of Chemistry). Tris-NiNTA-azide was first bound to the liposome using click chemistry. For this, Tris-NiNTA-azide was combined with DBCO liposome at a 2:1 molar ratio (100nmole and 50nmole, respectively) in 200μl and incubated overnight at RT to allow binding of the azide functional group to the DBCO phospholipid. Excess Tris-NiNTA-azide was removed by dialysis using a 10KDa cut-off membrane. HA proteins were then conjugated to Tris-NiNTA liposomes *via* hexahistidine tags. For this, 50μg HA (0.3nmole trimer or 0.9nmole monomer) was added to Tris-NiNTA-azide-DBCO liposomes and incubated overnight at 4°C with gentle rocking. This stoichiometry was previously optimized with 6 x His GFP as a model protein and demonstration that all the protein was bound to liposomes by CL-4B sepharose gel filtration chromatography. To quantify binding of HA, liposomes were resolved on SDS PAGE gels and silver stained (**Supplementary Figure S1**) and the HA concentration determined from a standard curve of serially diluted HA protein run on the same gel using NIH ImageJ software.

### Fluorescent Labeling of Hemagglutinins

HA monomers (Sinobiological) were conjugated to fluorescent dyes using the DyLight™ Microscale Antibody Labeling Kit (Thermo Fisher Scientific, Waltham, MA; cat # 22858) according to the manufacturer’s instructions. Briefly, lyophilized HAs were reconstituted in 100μL H_2_O (1mg/ml) and 8μL of 0.67M borate buffer added. H1, H5 and H7 were added to DyLight™ 405, 488 and 650 (cat #s 53021, 53025, 8543), respectively, vortexed gently and incubated at room temperature for 1h. Excess unbound dye was removed by gentle mixing with 108μL of Dye Removal Resin (centrifuged prior to mixing to remove the storage buffer) followed by centrifugation at 1000 x g to collect the labelled proteins.

### Formulations and Mouse Immunizations

AddaVax™ (squalene oil-in-water emulsion) was purchased from Invivogen Inc. (San Diego, CA), CpG 1018 (TLR9 agonists) were purchased from Invivogen and Integrated DNA Technologies (Coralville, Iowa), respectively. CpG-ODN were dissolved in sterile water at 1mM as stock. Monophosphoryl lipid A (MPLA, a TLR4 agonist) was purchased from Avanti Polar Lipids Inc. (Alabaster, AL). Since it has limited solubility in aqueous solution, MPLA was integrated into DOPG liposomes (an inert colipid) at 1:5 molar ratio. All animal work was approved by the UCI Institutional Animal Care and Use Committee (IACUC protocol #AUP-18-096) and by the Animal Care and Use Review Office (ACURO) of the U.S. Army Medical Research and Materiel Command (USAMRMC). The laboratory animal resources at UCI are Internationally accredited by the Association for Assessment and Accreditation of Laboratory Animal Care (AAALAC #000238). Female C57Bl/6 mice (7-10 weeks of age) were purchased from Charles River Inc., and housed in standard cages with enrichment. Each vaccine dose comprised 2.5μg each HA protein (or cocktail of 3 different HAs, total 7.5μg/mouse), 1nmole MPLA and 3nmole CpG-1018 in sterile PBS, and mixed with an equal volume of AddaVax™. Vaccines were administered *via* subcutaneous (s.c.) route (50μL, base of tail) under brief anesthesia with inhaled isofluorane/O_2_ mixture. In the majority of experiments, the vaccine was administered as a single dose, although one or more boosts were used to increase neutralizing antibody titers, as detailed in the results section. Mice were weighed daily for approximately 2 weeks and monitored for any changes in behavior or appearance. Plasma was collected at regular time points by facial vein bleed under anesthesia with inhaled isofluorane/O_2_, and at the experimental endpoint by cardiac puncture under terminal anesthesia.

### Protein Microarrays

HA protein microarrays were fabricated as previously described (52, 53). Briefly, lyophilized influenza proteins (Sino Biological Inc., Beijing, China) were reconstituted to a concentration of 0.1 mg/ml in phosphate-buffered saline (PBS) with 0.001% Tween 20 (T-PBS) and printed onto nitrocellulose-coated glass Oncyte^®^ Avid slides (Grace Bio-Labs, Inc., Bend, OR) using an Omni Grid 100 microarray printer (Genomic Solutions). The content of the array (**Supplementary Table S1**) comprised one or more variants of hemagglutinin (HA) subtypes 1-18 (both full-length HA0 and HA1 versions), neuraminidase (NA) subtypes 1-11, as well as representatives of influenza B. For probing, mouse plasma samples were diluted 1:100 in protein array blocking buffer (GVS, Sanford, ME, USA) supplemented with 10 mg/ml *E. coli* lysate (GenScript, Piscataway, NJ, USA) and 10μg/ml of amino acid peptide HHHHHHHHHHGGGG (Biomatik, Wilmington, DE) to block any anti-polyhistidine antibodies, and pre-incubated at room temperature (RT) for 30 min. Concurrently, arrays were rehydrated in blocking buffer (without lysate) for 30 min prior to replacement of the blocking buffer with pre-incubated sera. Arrays were incubated overnight at 4°C with gentle agitation. After washing 6 times with Tris-buffered saline (TBS) containing 0.05% Tween 20 (T-TBS) at RT, arrays were incubated in biotinylated-SP-conjugated goat anti-mouse IgG (Jackson Immunoresearch, West Grove, PA) diluted 1:200 in blocking buffer for 2h at RT. Arrays were then washed 6 times with T-TBS, followed by incubation with streptavidin-conjugated Qdot-800 (ThermoFisher) diluted 1:250 in blocking buffer for 1h at RT. Arrays were washed three times each with T-TBS followed by TBS, dipped in distilled water, and air-dried by centrifugation at 500 × g for 10 min. Images were acquired using the ArrayCAM imaging system from Grace Bio-Labs (Bend, OR) with gain and exposure times of 50 and 400-1000ms, respectively. Spot and background intensities were measured using an annotated grid (.gal) file. Signal intensities (SI) for each antigen on the array were first background corrected by subtracting sample-specific T-PBS buffer signals from purified protein spot signals.

### Avidity and Titer Measurements by Microarray

Avidity measurements were performed on protein microarrays with Na-thiocyanate (NaSCN) as chaotropic agent, based on methods widely used for ELISAs (54, 55). Briefly, arrays were probed with plasma in duplicate overnight as described above, then washed three times each with T-TBS followed by TBS. One array was incubated in 1.5M NaSCN for 15 mins at RT to elute low avidity antibodies, while the untreated array was incubated in PBS. After washing 6 times in T-TBS, both arrays were incubated in secondary antibody and tertiary reagent as described above. An avidity index was calculated from the SI for each antigen according to the following formula: ([SI after NaSCN treatment]/[SI of untreated]) x 100. No noticeable effect was seen on array profiles when samples were probed on arrays pre-treated with 1.5M NaSCN or PBS (data not shown) suggesting there was negligible effect of NaSCN on antigen integrity (56).

### Antigen-Specific B Cell Labeling and Affinity Measurements by Flow Cytometry

Mice were immunized s.c. with either individual H1, H5 or H7 antigens, or cocktails of each, as described above. For B cell-labeling, mice were boosted with the same formulation *via* the i.p. route 5 days prior to harvesting splenocytes. Single splenocyte suspensions (2 x10^6^ cells/well in 96-well round-bottom plates) were first surface labelled at room temperature (RT) for 30 mins with a cocktail of fluorescent Abs and reagents, comprising BV510-anti-CD138, BV655-anti-CD3, BV785-anti-CD19, PE-anti-IgG2a, PE/Cy-anti-GL7, and APC/Cy7-anti-IgM (BioLegend Inc., San Diego, CA) and 7-Aminoactinomycin D (7AAD; Thermo Fisher). After washing in PBS, cells were fixed, permeabilized and washed using an intracellular staining kit (Cyto-Fast™ from BioLegend) according to the manufacturer’s instructions. Next, the permeabilized and fixed cells were incubated in 100ng/well (50μL total vol) of fluorescently-labeled HA monomers, DyLight 405-H1, DyLight 488-H5, DyLight 650-H7 in Cyto-Fast Perm/Wash buffer for 1h at RT to label antigen-specific B cells. Cells were then washed in Perm/Wash buffer and the affinity of antibodies assessed by a ‘cold chase’ of 2.5μg/well unlabeled H1, H5 and H7 monomers for 1, 4 and 16 hrs. Finally, 100μL samples were acquired from 96-well plates on a Novocyte 3000 flow cytometer for 50 sec, with FSC-H threshold set at 100,000, and voltage settings such that unstained cells have autofluorescence values between 10^2^-10^3^ units. Unstained and single-color stained controls were used to set up the compensation matrix.

Flow data were analyzed with FlowJo software (FlowJo Llc., Ashland, OR). Live lymphocytes were gated by appropriate forward vs side scattering, followed by singlet gating by area vs. height of forward scattering pulse, and finally followed by 7AAD-gating of live (non-apoptotic and non-necrotic cells). CD19+ (including dim or lo and bright or high) cells were gated in a two-dimensional CD19 vs. CD3 plot, followed by gating of antigen-specific (plasma) cells in a 2D CD138 vs. CD19 plot (plasma cells are defined CD138+ CD19lo). For additional 2D plots, gated plasma cells were plotted in 2D showing H1 vs. H5, H1 vs. H7 and H5 vs. H7 to measure potential cross-reactivity. Additional statistics were carried out using tables and layouts in FlowJo followed by rendering graphs in Excel (Microsoft Corp., Redmond, WA).

### Hemagglutination Inhibition Assays

HI assays were performed essentially as described (57). Mouse sera samples were tested in duplicate by first treating 10μL sera with 30μL receptor destroying enzyme (Denka Seiken, Inc.) for 18h at 37°C, after which 30μL of 2.5% sodium citrate was added and heated at 56°C for 30 minutes (58). The volume was brought up to 100μL with PBS, pH7.2 to give a starting dilution of 1/10. Duplicate serial dilutions of 25μL sera/well were performed across a V-bottomed microtiter plate (Thermo Fisher) in HI assay buffer (FTA Hemagglutination Buffer, Fisher Scientific) and an equal volume of virus (diluted to 4 HAU/25μL HI assay buffer) added for 30 min at room temperature to allow neuralization to occur. One row without sera or virus (HI assay buffer only) and one row of without sera (virus only) were also included as controls. An equal volume of 0.5% freshly-washed turkey red blood cell suspension (Rockland Immunochemicals, Inc., Limerick, PA) in HA buffer was then added and left for 45 mins at 4°C to allow red cell pellets to form. The neutralization titer was defined as the reciprocal of the last dilution in which a clear pellet was seen, and multiplied by 10 to correct for the initial dilution from RDE treatment. An HI titer of 1/40 is considered the cut-off for protection in humans (59). Positive control sera for HI assays were produced against each virus in C57Bl/6 mice administered TCID_50_ of 5 x 10^4^ in 10μl *via* (intranasal) i.n. route followed 2 weeks later by the same dose administered in 100μl intraperitoneally (i.p.). TCID_50_ is the tissue-culture infective dose, i.e., dilution of virus that caused a cytopathic effect in 50% of wells containing MDCK cells (58).

### Data Analysis

For statistical analysis, a floor of 10 was set for array signal intensities and the data transformed by Log2. Vaccine groups were compared using two-tailed Mann-Whitney test for unpaired data and the two-tailed Kruskal-Wallis test with Dunn’s multiple-comparisons using GraphPad Prism 6.7 (GraphPad, La Jolla, CA, USA). A P value of <0.05 was considered statistically significant. To determine antibody breadth, geometric means of Log2-transformed data for each antigen were determined for each vaccine group, and an antigen was scored seropositive if above a cutoff defined as the mean+2SD of the signals for an irrelevant protein on the array (NA) which was not used to immunize. HA sequence alignments were performed with ClustalW and phylogenetic trees generated using MEGA 5.0 using the maximum likelihood method with a bootstrap analysis of 100. Separate analyses were performed on HA1 and HA2 after removal of the leader peptide and transmembrane sequences. Antibody titers were determined by performing eight 3-fold serial dilutions of plasma samples starting at 1/100. Sigmoidal curves were fitted to the data in GraphPad Prism and the dilution that gave a signal midway between the baseline and maximum (midpoint titer) determined using the IC50 function.

### Data Availability

Protein microarray data has been posted on the Genome Expression Omnibus (https://www.ncbi.nlm.nih.gov/geo/) under accession numbers GSE178932 and GPL30316.

## Results

**Figure 1:**
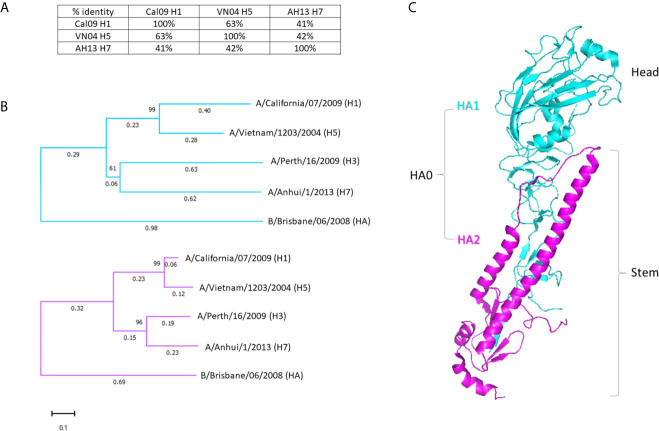
Hemagglutinins used in this study. **(A)** Percentages of amino acid identities of the three HA variants used for immunization: H1 from A/California/07/2009 (Cal09), H5 from A/Vietnam/1203/2004 (VN04), and H7 from A/Anhui/1/2013 (AH13). Percentages refer to the full-length (HA0) protein. **(B)** Dendrogram of separate HA1 and HA2 sequences of HA subtypes. HA from an additional Group 2 virus (H3, from A/Perth/16/2009) and a representative influenza B virus are included for context. HA1 sequences shown in cyan, HA2 sequences in magenta. **(C)** 3-dimensional representation of the HA monomer with HA1 and HA2 sequences colored as in **(B)** (PDB: 3ztn).

Protection against evolving pathogens remains a challenge for vaccination. Therefore, a major aim of this study was to evaluate the breadth of the antibody response produced by an adjuvanted variant antigen vaccine. As a secondary, aim we were interested to see if a similar breadth could be achieved using a single-dose regimen. As the model variant antigen in this study, we used influenza virus hemagglutinin (HA), represented in this study by single drift variants of subtypes H1 from A/California/07/2009 (“Cal09” H1), H5 from A/Vietnam/1203/2004 (“VN04” H5), and H7 from A/Anhui/1/2013 (“AH13” H7). Phylogenetic studies of HA subtypes have revealed two broad phylogenetic groupings: Group 1, which contains subtypes H1, H2, H5, H6, H8, H9, H11, H12, H13, H16, H17 and H18 and Group 2, which contains subtypes H3, H4, H7, H10, H14 and H15. Accordingly, Cal09 H1 and VN04 H5 (Group 1) share 63% sequence amino acid sequence identity overall, while Cal09 and VN04 are 41% and 42% identical with AH17 H7(Group 2), respectively (**Figure 1A**). The diversity between the HA sequences is non-uniform along the primary amino acid sequence, but is concentrated in the head domain of the molecule (**Figure 1B**). The variable head is encoded in the HA1 fragment of HA which also encodes part of the more conserved stalk domain (**Figure 1C**). In contrast, the HA2 region, which encodes the remainder of the stalk, is relatively well conserved between subtypes. The viruses in Group 1 (H1 and H5) and Group 2 (H3 and H7) cluster together when either the HA1 or HA2 sequences are aligned. Influenza B is clearly distinct from influenza A, with the HA1 sequences showing less sequence identity than HA2.

### Individual HA Proteins Engender Homo- and Hetero-Subtypic Cross-Reactivity *In Vivo*

We first measured the breadth of antibody profiles engendered by individual HAs, to provide a baseline for subsequent studies when the same antigens were administered as a multivalent vaccine. For this, C57Bl/6 (B6) mice were administered trimerized HA proteins in adjuvant and immune plasma assayed on HA protein microarrays to quantify the antibody cross-reactivity generated by each protein. In initial experiments, antigens were administered either in soluble form or coupled *via* poly-histidine tags to liposomes. Each was then formulated with or without an adjuvant comprising CpG (ODN 1018), MPLA, and a squalene-in-water emulsion, AddaVax™, a potent combination adjuvant we term ‘IVAX-1’. This particular adjuvant was chosen on the basis of a systematic screen of different TLR agonist combinations, (**Supplementary Figure S2**). In each case, the quantity of antigen loaded onto liposomes was determined by silver-stained sodium dodecyl sulphate polyacrylamide gel electrophoresis (SDS PAGE) gels by comparing the antigen band against a standard curve of known concentrations of antigen (**Supplementary Figure S1**). From this, the quantity of particles was adjusted so that each vaccine dose delivered 2.5ug of antigen per mouse.

Serological profiling of plasma at different time points post-immunization was determined using a custom HA protein microarray (53, 60) comprising 125 monomeric full length HA proteins (HA0) and 131 HA1 regions spanning 18 HA subtypes (**Supplementary Table S1**). In initial studies, Ab profiles were essentially identical regardless of whether the adjuvanted HA proteins were administered on solid or fluid liposomes, or as soluble antigen, (**Supplementary Figure S3**) which we speculate is caused by the liposomes being subsumed into the oil droplets of the emulsion. The antibody response was absolutely dependent on adjuvant, as none of the animals administered soluble antigen (HA alone) or liposome-bound antigen (both in the absence of IVAX-1 adjuvant) produced measurable antibody after a single dose (data not shown). Thus, in subsequent experiments, the HA trimers were administered as soluble proteins in the presence of IVAX-1 adjuvant only.

**Figure 2:**
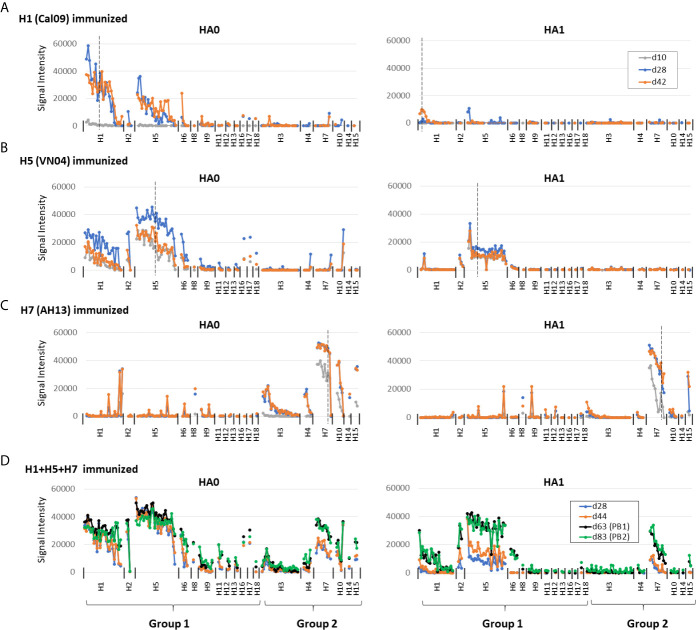
Administration of adjuvanted individual recombinant HAs induces homosubtypic and heterosubtypic cross-reactivity. **(A–C)** IgG profiles of C57Bl/6 mice at three time points (days 10, 28 and 42) after a single-dose s.c. administration of recombinant Cal09 H1, VN04 H5 or H13 H7 trimers, respectively, (2.5μg/mouse) in IVAX-1 adjuvant (comprising CpG, MPLA and squalene-in-water emulsion, AddaVax™; N=5 mice per group; **(D)** IgG profiles for C57Bl/6 mice (N=6) at four time points after administration of trivalent cocktail (2.5μg each) of Cal09 H1 + VN04 H5 + AH13 H7 in IVAX-1 adjuvant on d0, d50 and d72; plasma samples were collected on d28, d44, d63 (post-boost 1, PB1) and d83 (PB2). Profiles were determined using HA protein microarrays comprising 125 full length HA monomers (HA0, left side panels) and 131 HA1 regions (right side panels), spanning 18 different HA subtypes. Points are means of signal intensities for each vaccine group for each arrayed antigen. The ranking of antigens from left to right is the same in each panel: group 1 proteins are ranked by descending mean of H1+H5 immunized groups, while group 2 proteins are ranked by descending mean of H7 immunized mice. Vertical dashed line shows position of immunizing HA (or closest homolog). Animals that received antigen without adjuvant did not produce detectable IgG (data not shown). Data are representative of two independent experiments. See **Table 1** for numbers of seropositive antigens.

Shown in **Figure 2** are IgG profiles of mice administered a single dose of soluble H1, H5 or H7 trimers in IVAX-1 adjuvant. All three elicited Abs against the immunizing variant and against variants of the same subtype not present in the vaccine (homo-subtypic cross-reactivity). Thus, the response to H1 variants in mice administered Cal09 H1 was undetectable at d10 but reached a peak by d28 and remained elevated at d42. At its peak, Cal09 H1 elicited Abs to 18 of 24 (75%) full-length H1 variants, but only 2 of 23 (9%) HA1 variants on the array (**Figure 2A**; summarized in **Table 1**). Responses to VN04 H5 and AH13 H7 were quicker to develop. Thus, mice administered VN04 H5 showed detectable IgG responses on d10 that peaked on d28 but declined thereafter (**Figure 2B**). The profile was also broader, reacting with 26 of 26 (100%) full length H5, and 25 of 26 (96%) HA1 variants on the array. Similarly, mice administered AH13 H7 showed detectable IgG responses on d10 that peaked on d28 and remained elevated at d42 (**Figure 2C**). The H7 profile was also broad, reacting with 9 of 10 (90%) full length and 11 of 11 HA1 H7 variants.

**Table 1:** Summary of cross-reactivity profiles elicited by individual HAs.

Seropositive (%)				Cal09 H1 immunized		VN04 H5 immunized		AH13 H7 immunized	
		total HA0	total HA1	HA0	HA1	HA0	HA1	HA0	HA1
Group 1	H1	24	23	75	9	88	9	13	0
	H2	3	3	33	0	67	100	0	0
	H5	26	26	65	4	100	96	4	4
	H6	5	5	20	0	100	40	0	0
	H8	1	1	0	0	100	0	100	100
	H9	9	8	0	0	22	0	0	13
	H11	3	3	0	0	33	0	0	0
	H12	2	2	0	0	0	0	0	0
	H13	1	2	0	0	100	0	0	0
	H16	1	1	0	0	100	0	0	0
	H17	1	1	0	0	100	0	0	0
	H18	1	1	0	0	100	0	0	0
Group 2	H3	24	31	0	0	0	3	29	0
	H4	5	5	0	0	20	0	60	0
	H7	10	11	10	0	10	0	90	100
	H10	5	5	0	0	20	0	80	0
	H14	1	1	0	0	0	0	100	0
	H15	2	2	0	0	0	0	100	100
Other	B	13	7	0	0	0	0	14	15

Groups of mice (N=5 per group) were administered Cal09 H1, VN04 H5 or AH13 H7 in IVAX-1 adjuvant. Plasma was obtained at different time points and probed against a protein microarray (see **Figure 2**). Numbers of variants for each HA subtype printed on the array (both HA0 and HA1) are shown left. Heat map values are percentages of variants within each subtype that were seropositive on d28 or d42, whichever was greater. The cutoff for seropositivity was defined using Log2 transformed data as the average + 2 standard deviations of the signals against variants of a control antigen, neuraminidase (NA from A/Egypt/2321NAMRU3/2007 was excluded owing to producing consistently high false-positive signals). Proteins with murine Fc tags have been removed from the totals.

**Figure 3:**
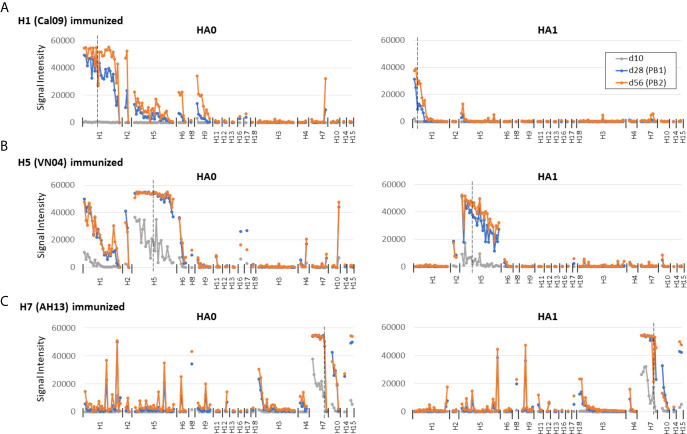
Prime-boost administration of individual HAs. For comparison with single-dose administration, C57Bl/6 mice were administered individual HAs in IVAX-1 as described in **Figure 2** and boosted on d14 and d42. Plasma samples were collected on d10, d28 (post-boost 1, PB1) and d56 (PB2). Panels **(A–C)** as per **Figure 2**.

The weak response to H1 HA1 may have been an artifact caused by conformation issues associated with H1 HA1. However, we identified two conformation-sensitive mAbs that bind H1 HA1 on the array, that lost reactivity against denatured antigen, suggesting the HA1 regions on the array are in a native configuration (**Supplementary Figure S4**). Moreover, the breadth of Abs against H1 HA1 increases to include all the 2009 variants after boosting (**Figure 3A**), and beyond the 2009 H1 HA1 variants after boosting with a trivalent cocktail (**Figure 2D**, as discussed below). Overall, these data indicate the weak response to H1 HA1 after a single dose H1 administration is because of reduced immunogenicity of H1, a notion supported by B cell labeling experiments below, rather than an artifact of the array.

Despite the robust signals on the protein arrays, plasma samples after a single dose of vaccine were non-neutralizing in an HI assay (data not shown) and required a boost (see below). Nevertheless, the data indicate administration of adjuvanted individual HA subtypes can engender broad cross-reactivity for drift variants within the same subtype, consistent with the sharing of conserved epitopes by different variants.

Responses to other HA subtypes not included in the vaccine (hetero-subtypic cross-reactivity) could also potentially contribute to protection by vaccination. The data after a single dose (shown in **Figure 2**) reveal hetero-subtypic cross-reactivity is generated to full-length (HA0) HA subtypes with the closest sequence identity to the immunizing antigen. For example, mice receiving Cal09 H1 cross-reacted with some variants of other group 1 subtypes, H2, H5, and H6, but not group 2 subtypes. It is interesting to note at this point that one mouse receiving Cal09 H1 mounted a robust response to H7 variants (a group 2 subtype), although this was atypical and was not seen in the other mice receiving Cal09 H1. Similarly, mice receiving adjuvanted VN04 H5 generated heterosubtypic cross-reactivity for several other group 1 subtypes, notably H1, H2, and H6. Mice administered adjuvanted AH13 H7 generated heterosubtypic cross-reactivity for other group 2 subtypes, notably H3, H4 and H10. Of interest, heterosubtypic cross-reactivity was confined mainly to the full-length HA0 proteins only and not HA1 regions, suggesting heterosubtypic cross-reactivity observed, at least from a single dose, is directed preferentially to the more conserved stem region of HA. Peculiar to H7 was cross-reactivity for a small number of variants of group 1 proteins, H1 and H5 (see **Figure 2C** and **Supplementary Figure S2C**). The pattern appeared stochastic and was unrelated to any obvious sequence identities, and the origins currently remain unclear.

The magnitude of heterosubtypic cross-reactive signals was consistently lower than the homosubtypic signals, and in some cases was slower to develop (see response to H7 compared to H3 in **Figure 2C**). This observation was confirmed in an independent experiment (**Supplementary Figure S5**) and is consistent with the notion that the strength of the cross-reactive response is related to sequence conservation between immunizing and cross-reacting Ag.

To place data obtained by single-dose administration into context, mice were administered a prime and two boosts of individual HAs in IVAX-1 (**Figure 3**). Overall, the magnitude of the response was higher after boosting than the single dose. In particular, it is noted that the weak response to the HA1 regions of H1 was expanded to comprise the 2009 variants on the array (**Figure 3A**).

**Figure 4:**
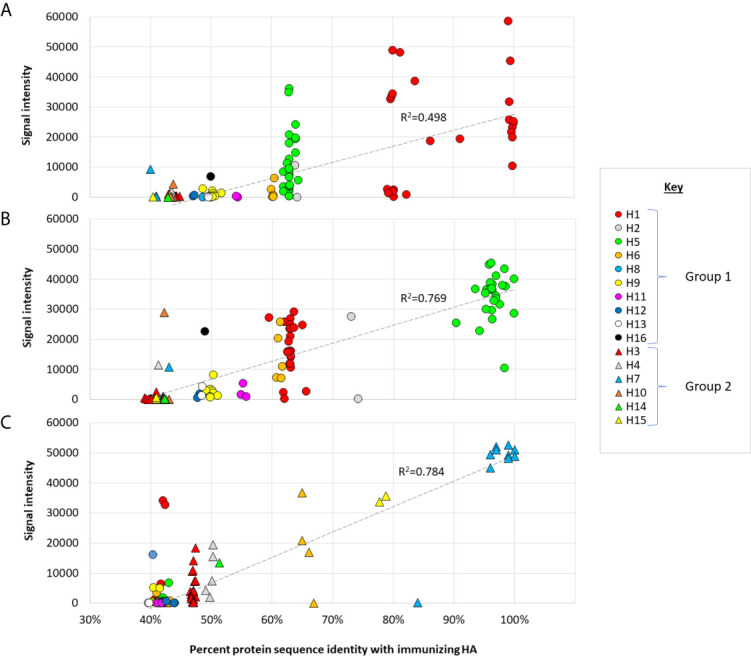
Cross-reactivity as a function of sequence identity. Scatter plots of IgG array signals after single-dose administration full-length **(A)** Cal09 H1; **(B)** VN04 H5; **(C)** AH13 H7, as shown in **Figure 2** (d28), plotted against % amino acid identity full-length proteins on the array. Circles, group 1 HAs; triangles, group 2 HAs. Hashed line, linear trend-line fitted through all data points.

The relationship between antibody reactivity and sequence relatedness between vaccine strain and detection antigen is examined further in the scatter plots shown in **Figure 4**.** **A clear trend is seen where the highest antibody signals are generated against homosubtypic variants of the immunizing Ag, followed by heterosubtypic variants in the same phylogenetic group, followed by heterosubtypic cross-reactivity for subtypes in the opposite phylogenetic group. Despite the trend, there are many variants that fall significantly above or below the line of best fit, indicating reactivity on the array (as measured by SI) is influenced by other factors in addition to sequence identity, such as Ag conformation and Ab titer.

### The Majority of Plasma Cells From Individual HA Immunizations Are Monospecific

**Figure 5:**
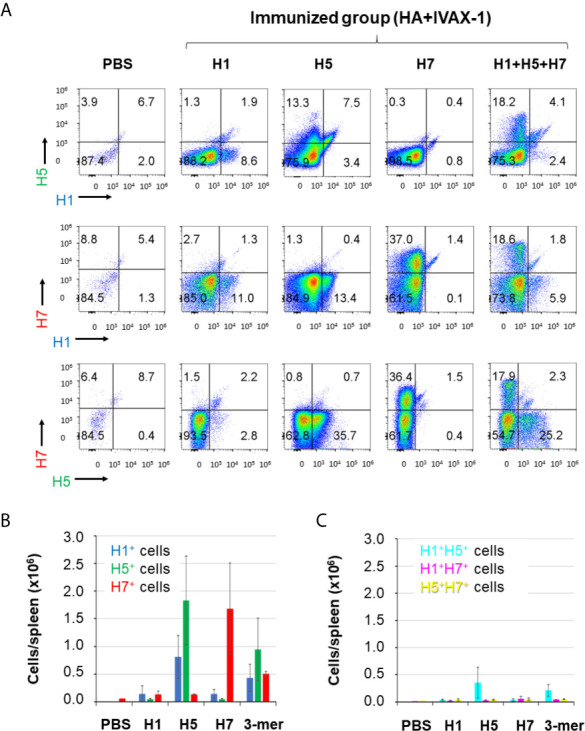
The majority of plasma cells from HA-trimer immunizations are antigen-monospecific. Mice were administered HA trimers individually (H1, H5 or H7; 3 μg per dose) or combined (H1+H5+H7), both in IVAX-1 adjuvant. For B cell labeling, mice were boosted on d45 i.p. with the same formulation, and sacrificed 5 days later. Single cell suspensions from individual spleens were surface-labelled with appropriate CD markers (see *Materials and Methods*), then fixed and permeabilized, and probed with HA monomers conjugated to fluorescent dyes to label antigen-specific B cells: H1-DyLight405, H5-DyLight488 and H7-DyLight650. **(A)** Representative plots of plasma cells (CD138+ CD19lo) from different vaccine groups, with H1, H5 or H7 fluorescence indicated on x- and y-axes. **(B)** Bar graph of the total number (+/- SD) of H1-specific, H5-specific and H7-specific plasma cells per spleen in mice immunized as in **(A)**. **(C)** Bar graph of the total number of dual-specific (H1+H5+, H1+H7+ and H5+H7+) plasma cells per spleen in mice immunized as in **(A)**. Data representative of several replicate experiments.

Array data presented above indicated that individual HAs are able to elicit cross-reactivity for other HA subtypes. It is also of interest to define the proportion of plasma cells producing HA subtype-specific *vs.* cross-reactive antibodies to underlie the serological data. Thus, we labeled antigen-specific plasma cells with H1, H5 and H7 proteins, each conjugated to a different fluorescent dye, and quantified antigen-specific and cross-reactive plasma cells by flow cytometry. Two-dimensional plots of antigen-specific plasma cells (CD138+ CD19lo) are shown in **Figure 5A** and as bar charts of total antigen-specific cells/spleen in **Figure 5B**. Antigen-specific plasma cells account for 8.6%-11% of all plasma cells in H1-immunized mice, 13.3%-35.7% in H5-immunized mice, and 36.4%-37% in H7-immunized mice. Mono-specific responses to a subtype other than used for immunization were at background levels. The relatively small number of H1-specific cells was consistent with the magnitude of the antibody response to H1 relative to other subtypes, as seen on microarrays, particularly against the HA1 region (see **Figure 2**). Interestingly, while the vast majority of plasma cells are HA subtype monospecific, there are smaller numbers of dual-specific plasma cells (i.e., H1+H5+, H1+H7+ and H5+H7+ plasma cells) (**Figure 5C**), indicative of cells with cross-reactive B-cell receptors (BCRs). Sequence identity between the Group 1 HAs, H1 and H5 (see **Figure 1**) predicts these two share a higher number of epitopes than with the Group 2 H7 and therefore should induce a higher frequency of cross-reactive B cells. Accordingly, in mice administered H1, more plasma cells were cross-reactive for H5 than H7 (1.9% and 1.3%, respectively). In mice administered H5, which we speculated earlier is more immunogenic than H1, the difference between cross-reactive plasma cells for H1 and H7 was more dramatic (7.5% and 0.7%, respectively). Mice administered H7, which showed weak cross reactivity for H1 and H5 on arrays (**Figure 2**) also showed only small percentages of cross-reactive plasma cells by the flow assay (1.4% and 1.5%, respectively). Overall, these data show only a small proportion of the activated plasma cells produce heterosubtypic cross-reactive antibodies. Also shown in **Figure 5** are antigen-specific B cells in mice administered the multivalent (H1+H5+H7) vaccine. These mice have monospecific cells against all 3 HA subtypes, as well as dual-specific cells. These data are important as they show that combining H1+H5+H7 into a multivalent cocktail did not compromise the induction of responses to the individual components. This phenomenon is explored further in the next section. It is interesting to note there are approximately half the numbers of H5 and H7-specific B cells after cocktail administration compared to individual administration, while H1-specific cells are elevated. Other effects of cocktail administration are examined in the following section.

### Combining H1+H5+H7 Reduces Response To HA1

We hypothesized that combining HA variants into a multivalent vaccine may elicit broader coverage than could be achieved by hetero-subtypic cross-reactivity elicited from a single variant alone. Thus, mice were administered a cocktail of Cal09 H1, VN04 H5 and AH13 H7 (2.5μg each per mouse, total 7.5μg) in IVAX-1 adjuvant, and plasma samples collected at various time points were probed on protein microarrays (**Figure 3D**). At both time points collected after the prime (d28 and d42), the magnitude of the response against the full-length HA0 variants was high, and comprised a combination of homo-and hetero-subtypic cross-reactive profiles elicited by the individual subtypes in the vaccine. Of note, heterosubtypic cross-reactivity beyond the vaccine was maintained. Thus, cross-reactive responses from H1 and/or H5 to other Group 1 HAs not in the vaccine, notably H2, H6 and H9, were preserved. Similarly, cross-reactive responses from H7 to other Group 2 HAs, notably H3, H4 and H10 were also preserved. Thus, the reduced numbers of antigen-specific B cells noticed previously (**Figure 5**) does not appear to impact the breadth of the response for full-length HA.

**Figure 6:**
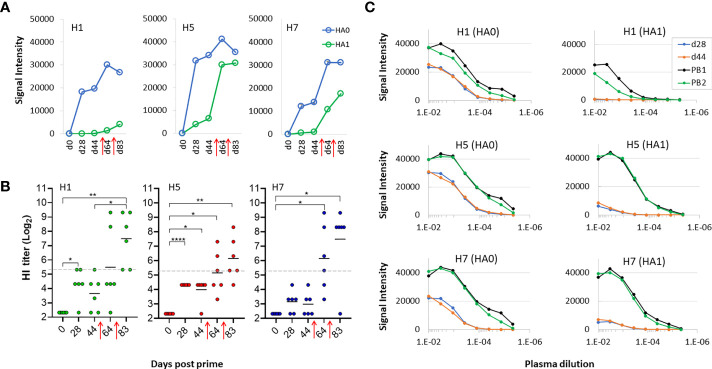
Boosting is required to generate virus neutralization after receiving trivalent vaccine. **(A)** means of signals at different time points for HA0 and HA1 variants of H1, H5 and H7 after administration of trivalent vaccine. **(B)** HI assay titers of individual sera used in panels **(A, B)** against PR8 reassortant influenza viruses expressing Cal09 H1, VN04 H5 and AH13 H7, respectively. Horizontal dotted line, 1/40 dilution cutoff (log-2 of 40 = 5.32) **(C)** serial dilutions of plasma from a representative mouse to determine titers at different time points using microarrays; shown are means of H1, H5 and H7 variants after serial dilutions. All data are representative of two separate experiments. PB1, post first boost; PB2, post second boost; red arrows, boosts. ****P < 0.0001; ***P ≤ 0.001; **P ≤ 0.01; *P < 0.05.

In contrast, the magnitude of the response to HA1 regions was lower after cocktail prime, compared to the corresponding HA0 responses. This phenomenon is emphasized in **Figure 6A** where the means of all H1, H5 and H7 HA0 and HA1 variants after multivalent administration plotted at different time points, showing lower signals against HA1 compared to HA0 after a single dose (d28 and d44). This suggests the response after a cocktail prime was dominated by antibodies to the stem, possibly because of their greater abundance compared to variable regions in the trivalent vaccine. These plasma samples also failed to neutralize in an HI assay, with titers below a 1/40 cut-off (log2 = 5.32) at d28 and d44 (**Figure 6B**), consistent with the weak response to HA1. After the mice were boosted with trivalent vaccine in IVAX-1 on d50 and again on d72 (red arrows) there was seen an increase the response to HA1, with a concomitant increase in HI assay titer. It is also interesting to note that boosting after cocktail administration expanded to H1 HA1 response to all of the HA1 (**Figure 6B**) These data suggest boosting with adjuvanted trivalent vaccine help to overcome any dominance of the stem when variants are combined, and allows neutralizing antibodies directed against the head to develop. The potential for driving the antibody response to the conserved stem by cocktail administration is an interesting phenomenon that warrants further study.

Since both full-length HA0 and HA1 possess the sialic acid binding site recognized by neutralizing Abs in the HI assay, it was of interest why only the array signals to HA1 increased after the boost. We suspected it is not readily seen on the array where the signals were already high. Therefore, Ab titers were determined by serial dilution on a subset of samples and probed on the array. Shown in **Figure 6C** are serial dilutions of plasma from a representative mouse at each time point. These data show that the titer of Abs against HA0 is ~10^-3^ prior to the boost and increases ~10-fold after the boost. In contrast, titers against HA1 are low before the boost and increase by several logs after the boost. Increasing the dose from 2.5μg of each HA subtype to 10μg did not overcome the requirement for boosting to engender neutralizing titers (data not shown). Overall, we conclude the multivalent approach engenders neutralizing activity to all 3 constituent HA subtypes although this required boosting to reach titers that can be detected in the HI assay.

**Figure 7:**
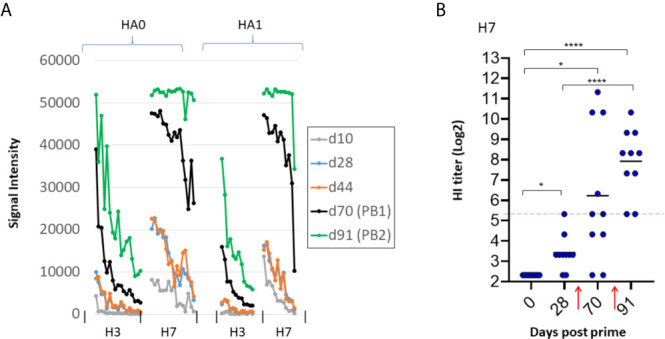
Higher antigen dose overcomes bar to induction of heterosubtypic cross-reactivity. B6 mice (N=10) were administered high dose trivalent vaccine (10μg each of Cal09 H1 + VN04 H5 + AH13 H7) in IVAX-1 adjuvant on d0; H7 cross-reactivity for H3 (not present in the vaccine) appears after boosting. **(A)** IgG profiles of H7 (present in the vaccine) and H3 (not present in the vaccine) at different time points. H3 signals (hetero-specific cross-reactivity) are elevated after boosting. Contrast with lower dose in **Figure 6** where no elevation was seen after boosting. **(B)** HI assay titers of individual plasma used in **(A)** against PR8 reassortant influenza virus expressing AH13 H7 shows boosting is required for appearance of HI activity. PB1, post first boost (d70); PB2, post second boost (d91). red arrows, boosts. ****P < 0.0001; ***P ≤ 0.001; **P ≤ 0.01; *P < 0.05.

We noted in the previous experiment that administration of second and third doses of trivalent vaccine enhanced hetero-subtypic cross reactivity to both Group 1 and Group 2 subtypes non present in the vaccine. However, boosting did not elevate the response to H3 which we know from **Figure 2** is cross-reactivity from the H7 component in the vaccine. We speculate this lack of boosting to H3 is a sequence dependent phenomenon, as H7 has lower sequence identity with H3 compared to other Group 2 antigens (see **Figure 3**). We found this bar could be overcome at a higher vaccine dose. Thus, mice were administered a trivalent vaccine comprising 10μg of each HA subtype (total 30μg/mouse) in IVAX-1 adjuvant, followed by two boosts. As expected, given the higher dose, array signals (Ab titers) were elevated before and after the boosts compared to the lower dose (2.5μg) used previously (data not shown). Importantly, the heterosubtypic cross-reactive signals against H3 increased after the boost (**Figure 7A**), which was not seen at the lower dose used previously. These data are consistent with the notion that heterosubtypic cross-reactivity is a function of both sequence identity and dose, wherein Abs to proteins with lower sequence identity can be engendered if administered at sufficiently high concentration. As before, boosting was still required to generate detectable levels of H7 virus neutralization in the HI assay (**Figure 7B**), illustrating the array detects both neutralizing and non-neutralizing antibodies.

### Combining H1+H5+H7 Does Not Compromise Maturation of the Response to Individual Components

**Figure 8:**
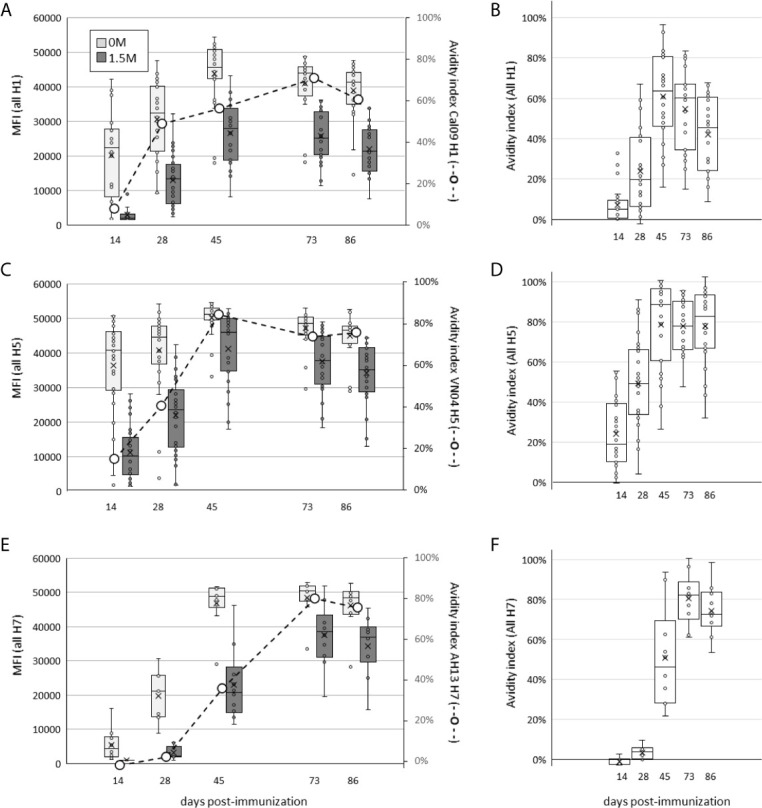
Administration of H1+H5+H7 multivalent cocktail engenders increases in antibody avidity to individual components over time. Mice were administered a single dose of trivalent cocktail in IVAX-1 adjuvant, and plasma samples collected at days 14, 28, 45, 73 and 86 post-immunization. Shown are array signal intensities for variants of the immunizing subtypes: **(A, B)**, H1 antigens; **(C, D)**, H5 antigens; **(E, F)**, H7 antigens. **(A, C, E)** show HA protein array signal intensities with or without 1.5M NaSCN (dark grey and light grey boxes, respectively); each panel is overlaid with avidity indexes of antibodies to the immunizing antigen (secondary axis, hashed line); **(B, D, F)** show corresponding avidity indexes to all H1, H5 and H7 variants, respectively, on the array.

We first examined the effect of multiplexing HA subtypes on the avidity of the polyclonal response, a function of both titer and affinity, using chaotropic dissociation. Animals receiving a single dose of H1+H5+H7 in IVAX-1 adjuvant showed a steady increase in magnitude of IgG signal intensity (SI) against components over time (light grey bars in **Figures 8A, C, E**, respectively), which peaks ~d45 and declines gradually thereafter. Overlaid onto the SI plots are avidity indices to the immunizing HAs (hashed line), which are all seen to increase with time after administration of the multivalent formulation. Furthermore, **Figures 8B, D, F** show the avidity of homosubtypic cross-reactive antibodies also increase over time. These data show combining H1+H5+H7 leads to avidity maturation to each of the individual constituents.

**Figure 9:**
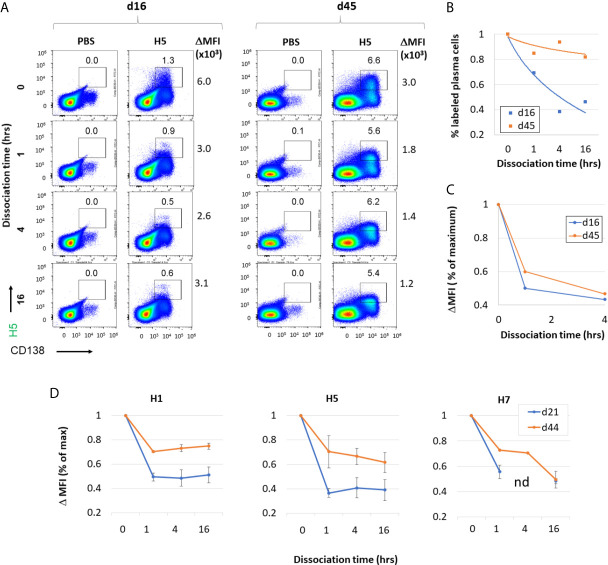
Administration of H1+H5+H7 multivalent antigen cocktail results in increased antibody affinity to individual antigenic components over time. **(A)** Validation of affinity maturation assay using vaccine containing H5 alone (2.5μg VN04 H5 trimer in IVAX-1) administered by s.c. route. Representative flow cytometry of plasma cells (x-axis) and H5(DyLight-488)-binding cells (y-axis) of unimmunized (PBS), or H5-immunized mice boosted on day 16 or 45 i.p. with the same formulation and splenocytes harvested 5 days later. Competition for binding with an excess of unlabeled H5 protein was performed for 0, 1, 14 and 16 hrs. This demonstrates specificity of H5 binding, as well as providing a measure of antibody affinity for H5 antigen according to rate of dissociation. **(B)** The % of H5-specific plasma cells, and **(C)** ΔMFI, defined as the difference in mean fluorescence intensity between the antigen-positive population (rectangular gate) and the antigen negative population (not shown). The experiment was performed independently on two mice at each time point. **(D)** ΔMFI of plasma cells positive for H1(DyLight-405), H5(DyLight-488), and H7(DyLight-650) in mice administered a trivalent formulation (2.5 μg each of H1, H5 and H7 in IVAX-1 adjuvant) *via* s.c route. Competition for binding with an excess of unlabeled H1, H5 or H7 was performed for 0, 1, 4 and 16 hrs. nd, not done.

We next examined affinity maturation using a flow-based assay to quantify affinity of antigen for B cell-receptors (BCRs). Antigen-specific plasma cells were labelled with fluorescent antigen, and then incubated with unlabeled antigen for different times to compete with fluorescent antigen. The loss of fluorescent label over time (i.e., dissociation rate) is a correlate of antibody affinity. The plots shown in **Figure 9A** show validation of the method using H5-immunized mice as an example. The percentage of H5-specific B cells labelled (boxed) decreases with increased time of incubation (0-16 h) with unlabeled H5 competitor. When the percentages of labeled plasma cells at different times incubation were normalized by expression as a % of maximum, the decrease is seen to be more rapid at d16 post immunization compared to d45 (**Figure 9B**). Similarly, when the ΔMFI (difference in MFI between the vaccinated and PBS vaccinated controls) was expressed as a % of maximum, the decrease with increased time incubation was also more rapid at d16 compared to d45 (**Figure 9C**). These data show an increase in affinity of H5-specific B cells with time after administration H5 vaccine. Similar cold-competition experiments with unlabeled H1 and H7 also showed antibodies to these also underwent affinity maturation after administration of H1 and H7 vaccines (data not shown). Shown in **Figure 9D** are ΔMFI data from mice receiving multivalent vaccine (H1+H5+H7 with IVAX-1 adjuvant) followed on d16 and d45 by measurement of affinity of H1, H5 and H7-specific B cells in the spleen. In each case there is a more rapid decrease in signal at the earlier time point. Overall, these data are consistent with the notion that combining H1+H5+H7 into a multivalent vaccine does not compromise affinity maturation to each of the constitutive components.

## Discussion

Antibody profiling studies, such as by microarrays as used here, have been facilitated in recent years by the availability of large numbers of recombinant HA variants for serological screening (13, 60–64). In this study, we measured antibody responses to mono and multivalent influenza hemagglutinin (HA) vaccines using HA protein microarrays, flow cytometry and neutralization assays, with the aim of understanding the requirements for inducing broad reactivity. Arrays revealed the administration of individual recombinant HA subtypes induces both antigen-specific antibodies against the immunizing antigen, and cross-reactive antibodies to other HA proteins not in the vaccine. The profiles of cross-reactive antigens were dominated by drift variants within the same HA subtype as the vaccine (homosubtypic cross-reactivity), as well as significant heterosubtypic cross-reactivity for other, closely-related subtypes (i.e., H5-induced antibodies cross react with H1 and vice versa, and H7-induced antibodies cross-react with H3). These data are consistent with related studies that show the strongest HA cross-reactivity occurs with variants of the highest sequence identity, which is progressively diminished by decreasing homology (61). Homosubtypic cross-reactivity was seen against both full-length and HA1 versions, while heterosubtypic cross-reactivity was confined to the full-length only, suggesting the latter was directed against the more conserved stalk. Flow cytometry of antigen-specific B cells confirmed the existence plasma cells with dual specific BCRs which would mediate the heterosubtypic cross-reactivity seen by array, although these were rare among total plasma cells.

We also compared the response to individual HAs against a multivalent vaccine (H1+H5+H7) approach for generating a broadly-reactive vaccine. The antibody response to the multivalent vaccine can be viewed as the sum of distinct, but partially overlapping, cross-reactivity profiles to the individual components. The main findings here were that combining the antigens did not appear to compromise the maturation of the response to the individual constituents, as measured by increases in titer or avidity by microarray, or affinity as determined by flow cytometry. Interference with affinity maturation is one potential caveat that has been raised against the deployment of variant antigen cocktails, based on coadministration of HIV gp160 variants (65). We did notice the response to the multivalent vaccine was skewed toward full-length HA0 variants on the array, with a weaker response to the corresponding HA1 regions. However, this was readily overcome after boosting, and there is a concomitant increase in HI assay titers. We also provide evidence that heterosubtypic cross-reactivity for more distantly related subtypes may be enhanced at higher antigen dose.

The potential for driving the antibody response to the conserved stem by cocktail administration is an interesting phenomenon that warrants further study, since this usually requires stem-directed immunization strategies such as chimeric HA or headless HA approaches (discussed below). It is possible combining variants drives the response to the conserved regions of the protein because these are represented at 3-fold higher concentrations than the variable sequences specific to each HA subtype. A similar broadening of the response is seen in other systems. The response to the variant apical membrane antigen-1 (AMA-1) antigen from *Plasmodium falciparum*, for example, can be broadened beyond that of the individual vaccine components when administered as a polyvalent vaccine (66–69). One study reports a minimum of three variants are required for the effect, and proposes that combining variants results in dilution of strain-specific epitopes relative to conserved epitopes, a phenomenon called ‘epitope dilution’ (70).

Combining variant antigens into a multivalent vaccine represents the traditional approach to achieve broad coverage against a variant pathogen, and is the basis of several approved vaccines against divergent viruses, including human papillomavirus (HPV), rotavirus, and poliovirus. Indeed, the tri- or quadrivalent inactivated ‘split’ influenza virus seasonal vaccines are the prototypic multivalent variant antigen vaccine and have been in use for the past 50 years. A universal influenza vaccine, designed to confer anticipatory protection to drift variants before they emerge, would overcome the need for seasonal re-vaccination (71). Broadening of the response beyond the vaccine is a hallmark of a universal influenza vaccine, and is likely to be best achieved by focusing the immune response to epitopes shared between different influenza strains, such as the stem or conserved regions in the head. However, for reasons that are not well understood, the variable head domain of HA is immunodominant over the more conserved stem, and antibodies produced during seasonal influenza infection (26) or after vaccination (27–30) are typically head-specific and not broadly reactive. Approaches to shift immunodominance from the head to the stem include sequential immunization of different variants to boost the response to conserved regions in the stem (16, 17), administration of “headless” HA molecules (18, 19, 72, 73), or conserved polypeptides (20), or by using adjuvants (see below). Nanoparticle delivery also assists with inducing broad reactivity. For example, cocktails of nanoparticle displaying HA from H1, H3, H5 and H7 elicited protection in mice against the immunizing subtypes as well as other subtypes not in the vaccine, including H2, H6, H7, H10, and H11 (74). Display of different variants on the same (mosaic) particle also induces a broad antibody response (75).

An additional strategy to induce cross-reactivity is to exploit pre-existing memory B cells that produce broadly cross-reactive antibodies. Conventional seasonal vaccines fail to boost responses to the stem (27). However, boosting with novel or “exotic” HA subtypes (i.e., not seen previously in human populations) preferentially stimulates pre-existing memory B cells that cross-react. This was first noticed during the 2009 H1N1 pandemic when the specificity of B cells in patients was determined at the single cell level for the first time. Contrary to expectations, the head was not immunodominant. Instead the majority of neutralizing antibodies were broadly cross-reactive for multiple influenza strains (76) and produced by pre-existing memory B cells to other influenza strains (77). Many broadly-reactive mAbs have since been cloned from influenza infections, the majority of which recognize conserved regions in the stem, with a smaller number targeting the head (78–81). This phenomenon, first revealed by the pandemic H1N1 virus, has since been recapitulated by sequential administration of chimeric HA molecules comprising a conserved H1 stem with different ‘exotic’ heads (82), and by use of an H5 vaccine (27).

In this study, we used a potent combination adjuvant consisting of CpG, MPLA and AddaVax™ emulsion throughout. Both CpG and MPLA are approved for use as adjuvants in humans (40–42). AddaVax™ is essentially identical to MF59^®^, a squalene-in-water emulsion that is approved for pandemic influenza vaccines (83, 84). Although traditionally used alone, recent studies have shown that combining adjuvants (so-called ‘combination adjuvants’) more accurately replicate the effects of natural infection (45), including long-lasting immunity and induction of broadly neutralizing antibodies. For vaccines, this translates into more durable protection, dose-sparing, and the requirement for fewer booster immunizations. In influenza, combination adjuvants have been shown to enhance homo- and heterosubtypic cross-reactivity beyond that seen with single adjuvants. For example, MPLA and CpG combination adjuvants enhance the protective efficacy in mice against homosubtypic and heterosubtypic viruses (46, 47). Other than those developed especially for the elderly (85), adjuvants are not routinely used for seasonal influenza vaccines, and are consequently weakly immunogenic and typically show very limited cross-reactivity. Adjuvants, notably TLR-agonists, both increase immunogenicity and breadth of response to inactivated seasonal vaccines (86, 87). Emulsions such as MF59^®^ and AS03 have also been reported to broaden the response of seasonal vaccines (27, 88–90). Uptake of adjuvants has also been limited for recombinant protein-based seasonal vaccines. FluBlok^®^, an FDA-approved is a tri- or quadrivalent seasonal influenza vaccine comprising recombinant HA from H1N1, H3N2 and one or two B HAs produced in insect cells, and is administered without adjuvant (91).

In contrast, adjuvants have been embraced for pandemic influenza vaccines for increasing immunogenicity and to reduce response times, with the added benefit of increased breadth of response (89) (83). For example, when used with H5N1 vaccines, the approved emulsion MF59 elicits both homo-subtypic (cross-clade) cross-reactivity for other H5 variants as well as hetero-subtypic cross-reactivity for H1 (85, 89, 92, 93). Similar results were observed with emulsion AS03 (94).

In summary, we conclude firstly, that cocktail administration of influenza HAs in adjuvant does not seem to impair the response to the individual components. This is a concern that has been raised in other systems, such as HIV for example. Second, the use of adjuvant with recombinant protein vaccine leads to a broad response beyond the content of the vaccine. The adjuvanted multivalent vaccine approach, which generates distinct but partially overlapping cross-reactivity profiles, may offer a more practical route to achieve broad coverage that is more achievable than using single variants.

## Data Availability Statement

The data presented in the study are depositied in the Genome Expression Omnibus (https://www.ncbi.nlm.nih.gov/geo/) under accession numbers GSE178932 and GPL30316.

## Ethics Statement

The animal study was reviewed and approved by the UCI Institutional Animal Care and Use Committee (IACUC protocol #AUP-18-096) and by the Animal Care and Use Review Office (ACURO) of the U.S. Army Medical Research and Materiel Command (USAMRMC).

## Author Contributions

JH-D, JF, EP, and DHD designed the experiments. JH-D, JF, SS, EP, AaJ, SJ, RN, AlJ, and DHD performed experiments or supplied reagents. JH-D, ES, and DHD performed data analysis and prepared figures. FK, PF, and DHD provided funding. JH-D and DD wrote the manuscript. All authors contributed to the article and approved the submitted version.

## Funding

Supported by Defense Threat Reduction Agency grant HDTRA1-18-1-0036. Work in the Krammer laboratory was supported by NIAID Collaborative Influenza Vaccine Innovation Centers (CIVIC) contract 75N93019C00051.

## Disclaimer

The views expressed in this article are those of the authors and do not reflect the official policy or position of the U.S. Department of Defense or the U.S. Army.

## Conflict of Interest

JH-D, JF, EP, AaJ, SJ, RN, AlJ, PF, and DHD own shares in Nanommune Inc. Nanommune does not sell any products described in this paper, nor funded any part of the work described herein. Neither Nanommune nor its shareholders are likely to benefit from the results described in this publication.

The remaining authors declare that the research was conducted in the absence of any commercial or financial relationships that could be construed as a potential conflict of interest.
