# Erratum: Administration of Multivalent Influenza Virus Recombinant Hemagglutinin Vaccine in Combination-Adjuvant Elicits Broad Reactivity Beyond the Vaccine Components

**DOI:** 10.3389/fimmu.2021.754535

**Published:** 2021-08-20

**Authors:** Frontiers Production Office

**Affiliations:** Frontiers Media SA, Lausanne, Switzerland

**Keywords:** vaccine, influenza, adjuvant, CpG, MPLA, ADDAVAX^®^, hemagglutinin

Due to a production error, there was a mistake in **Figure 6** as published. Part of the y axis title and scale in **Figure 6B** was obscured by a white area.

The corrected **Figure 6** appears below. The publisher apologizes for this mistake.

**Figure 6:**
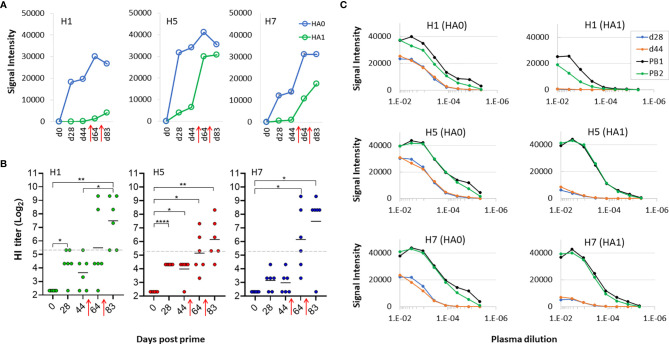
Boosting is required to generate virus neutralization after receiving trivalent vaccine. **(A)** Means of signals at different time points for HA0 and HA1 variants of H1, H5 and H7 after administration of trivalent vaccine. **(B)** HI assay titers of individual sera used in panels **(A, B)** against PR8 reassortant influenza viruses expressing Cal09 H1, VN04 H5 and AH13 H7, respectively. Horizontal dotted line, 1/40 dilution cutoff (log-2 of 40 = 5.32) (C) serial dilutions of plasma from a representative mouse to determine titers at different time points using microarrays; shown are means of H1, H5 and H7 variants after serial dilutions. All data are representative of two separate experiments. PB1, post first boost; PB2, post second boost; red arrows, boosts. ****P < 0.0001; ***P ≤ 0.001; **P ≤ 0.01; *P < 0.05.

The original version of this article has been updated.

