# Supplementary material for: Administration of Multivalent Influenza Virus Recombinant Hemagglutinin Vaccine in Combination-Adjuvant Elicits Broad Reactivity Beyond the Vaccine Components
Source: Front Immunol. 2021 Jul 14;12:692151. doi: 10.3389/fimmu.2021.692151 (PMC8318558; doi:10.3389/fimmu.2021.692151)
Supplement: Supplementary file 1 [file DataSheet_1.pdf]

Supplementary Figures

A) Liposome composition

Lipid name	MW	Fluid Liposome (Unsaturated Lipids) Mole %	Fluid Liposome (Unsaturated Lipids) mM	Solid liposome (Saturated lipids) Mole %	Solid liposome (Saturated lipids) mM
DOPC	786	92	18.4		
DOPG	797	5	1		
DSPE-PEG2000-DBCO*	3078	2.5	0.5	2.5	0.5
DOPE-Rhodamine**	1302	0.5	0.1	0.5	0.1
DPPC	734			63	12.6
DPPE-PEG750	1472			4	0.8
CHOL	387			30	6

* *For clicking to Azide-trisNTA*
** *Fluorescent label*

B) Tris-NiNTA-Azide

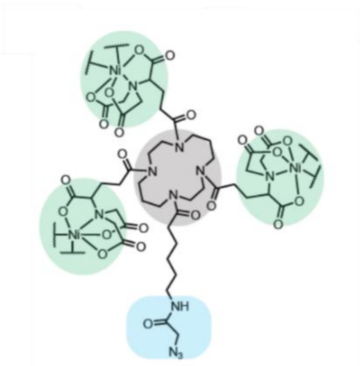

C) Heated at 95°C, 5 min

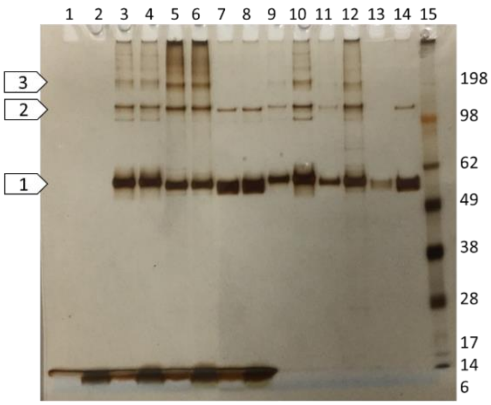

1	Fluid Liposome
2	Solid liposome
3	Fluidliposome-H1
4	Solid liposome-H1
5	Fluidliposome-H5
6	Solid liposome-H5
7	Fluidliposome-H7
8	Solid liposome-H7
9	H1 std 100ug/ml
10	H1 std 300ug/ml
11	H5 std 100ug/ml
12	H5 std 300ug/ml
13	H7 std 100ug/ml
14	H7 std 300ug/ml
15	Protein marker

D) No heat

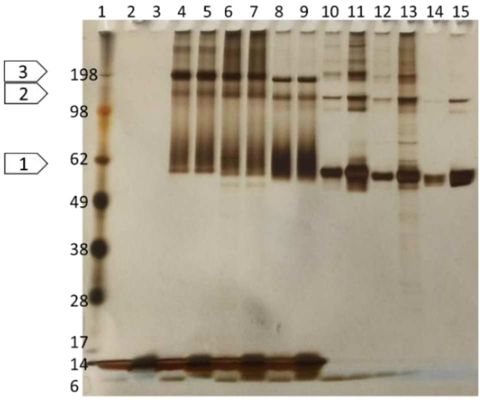

1	Protein marker
2	Fluid Liposome
3	Solid liposome
4	Fluidliposome-H1
5	Solid liposome-H1
6	Fluidliposome-H5
7	Solid liposome-H5
8	Fluidliposome-H7
9	Solid liposome-H7
10	H1 std 100ug/ml
11	H1 std 300ug/ml
12	H5 std 100ug/ml
13	H5 std 300ug/ml
14	H7 std 100ug/ml
15	H7 std 300ug/ml

Supplementary Figure S1. QC of HA-conjugated liposomes. a) Table of liposome compositions. See Materials and Methods for details. **b)** Schematic of the Tris-NiNTA-azide molecule (Mol. Wt. = 1,132) used to conjugate proteins with poly-His tags to DBCO functional groups on the liposome surface. **c)** and **d)** silver-stained SDS PAGE gels for conjugated liposomes with or without heat treatment, respectively, before resolving on 4-12% Bis-Tris precast gels using the MOPS buffer system for 60 minutes at 150V. Arrows indicate positions of monomers, dimers and trimers of HA (labeled 1, 2 or 3, respectively). Lanes in each gel are indicated in the accompanying tables. Expected molecular weights: HA monomer (556 amino acids) = 61kDa; HA dimer = 122kDa; HA trimer = 183kDa.

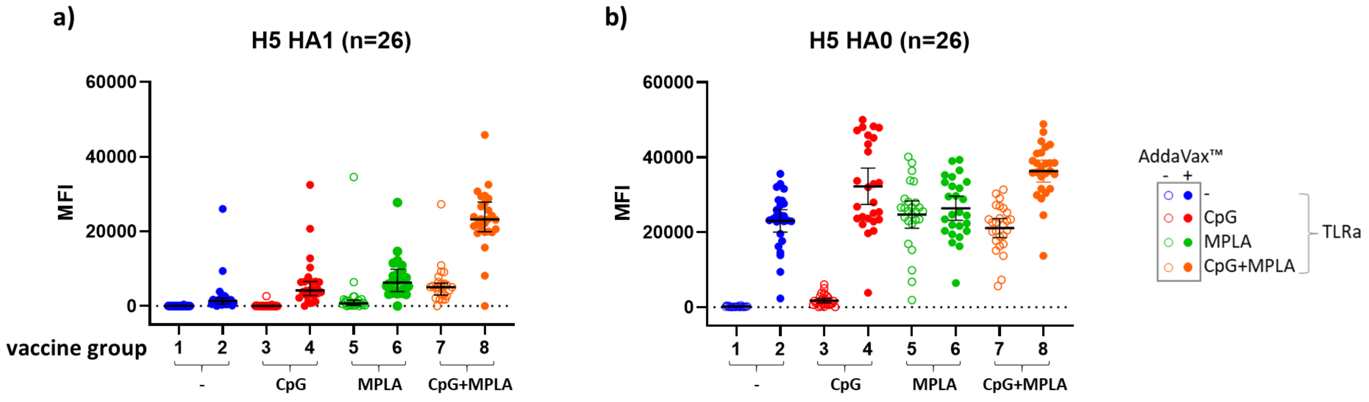

Supplementary Figure S2. Adjuvant screen. A single dose of VN04 H5 trimers was administered to C57Bl/6 mice (2.5μg/mouse; N=5 mice per group) with different TLR agonists (TLRa), without or with AddaVAX™ (open and solid symbols, respectively). Each spot represents a single H5 variant on the protein microarray (MFI = mean of 5 mice), represented as **(a)** HA1 regions, or **(b)** full-length HA0 proteins. Each plot is overlaid with median and interquartile range. Data shown are IgG signals for d28 post single-dose immunization. Similar data were seen using Cal09 H1 or AH13 H7 (not shown). The combination adjuvant CpG + MPLA + AddaVax™ (termed "IVAX-1") was used for subsequent studies on the basis of superior magnitude and breadth of response for both HA0 and HA1 proteins.

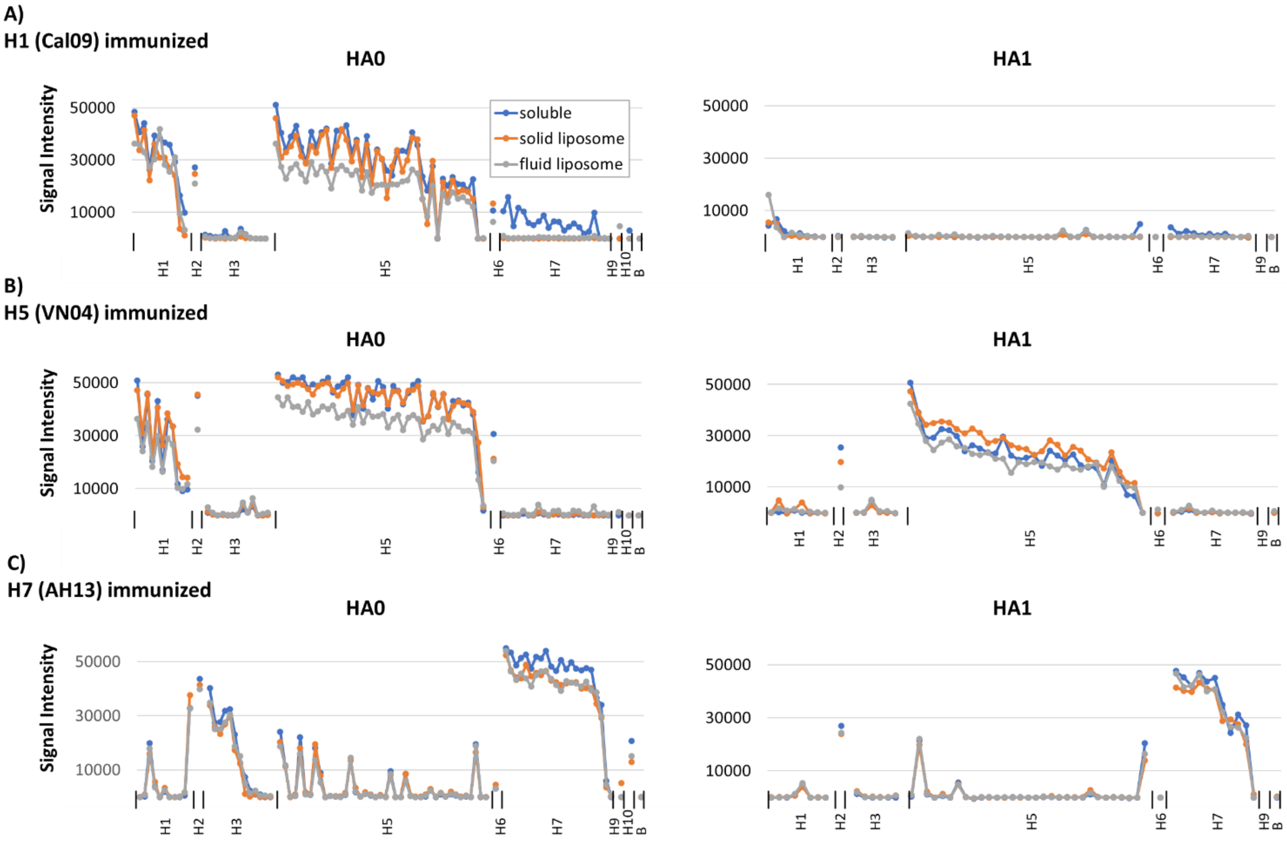

Supplementary Figure S3. IgG profiles elicited to individual HAs delivered as soluble trimers, or as trimers conjugated to solid or fluid liposomes, all administered in IVAX-1 adjuvant (CpG+MPLA+AddaVax™). Shown are signals for day 43 post prime, although similar data are seen on d28. Plasma samples were probed against an earlier iteration of the HA protein microarray that emphasized H1, H3 and H5 content.

a)

BEI Cat #	clone	Antigen
NR-28665	CA09-02	Cal09 H1
NR-28666	CA09-09	Cal09 H1
NR-28667	CA09-11	Cal09 H1
NR-28668	CA09-15	Cal09 H1
NR-42019	H1CA 5C12	Cal09 H1
NR-42021	H1CA 4F8	Cal09 H1
NR-19866	S-OIV-SH7	Cal09 H1
NR-13453	39E4	1918 H1
NR-13452	6B9	1918 H1

b)

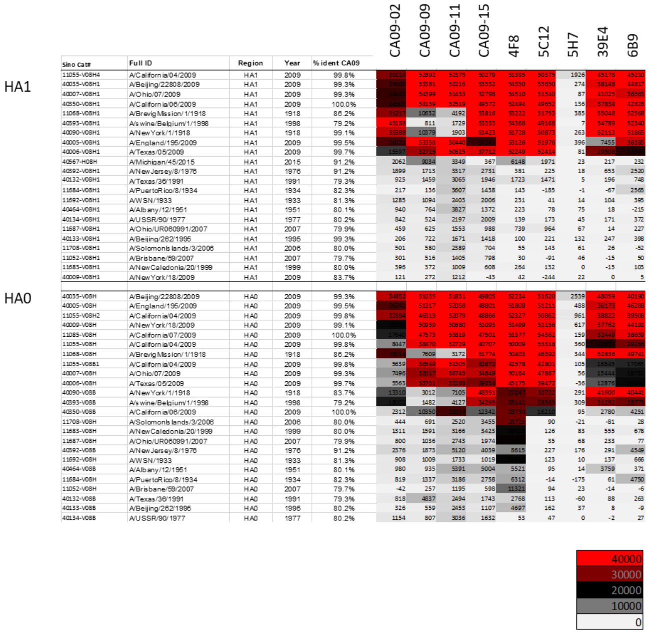

c)

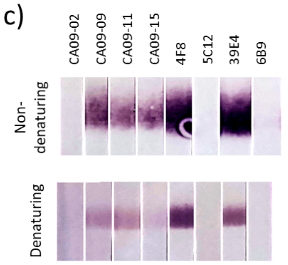

d)

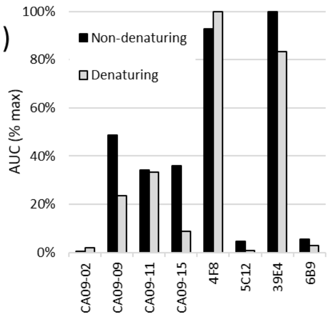

Supplementary Figure S4. Screen for conformation-dependent mAbs. **a)** The panel of 9 mAbs raised against A/Cal/2009 H1 (N=7) or 1918 H1 (N=2) which neutralize in HI assays (BEI Resources); **b)** Protein microarray of H1 drift variants, represented as full-length HA0 and HA1 fragments; **c)** Western blots of Cal09 H1 resolved on denaturing and non-denaturing SDS PAGE gels and probed with the 8-array reactive mAbs; **d)** band intensities of blots shown in c) quantified using ImageJ, and areas under the curve (AUC) converted to % max. MAb CA09-15 lost activity on denaturing gels (indicative of a conformation sensitive epitope), while CA09-09 also partially lost activity. Both mAbs recognize both HA0 and HA1 of the 2009 H1 variants on the array.

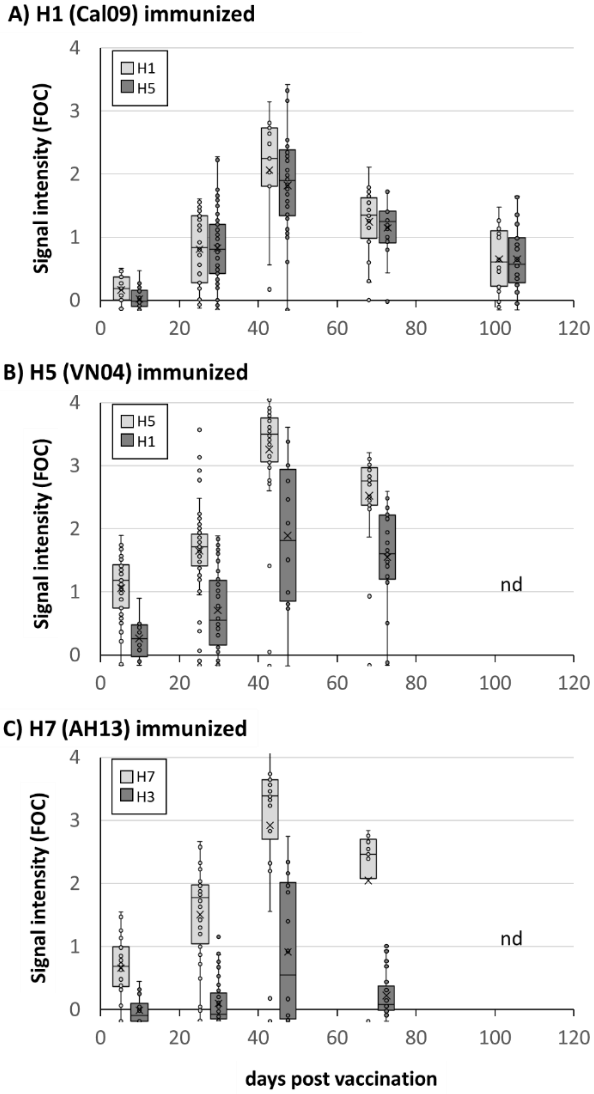

Supplementary Figure S5. Homosubtypic cross-reactivity develops more quickly and reaches higher signals that heterosubtypic cross-reactivity. Sera collected at different time points (days 10, 28, 43, 72 and 103) after administration of individual H1, H5 or H7 in IVAX-1 adjuvant were probed on HA arrays. Each data point represents the mean signal for each full-length (HA0) HA variant. **a)** reactivity of sera from H1-immunized mice for variants of H1 and a related group 1 HA, H5; **b)** reactivity of sera from H5-immunized mice for variants of H5 and H1; **c)** reactivity of sera from H7-immunized mice for variants of H7 and a related group 2 HA, H3. Light grey boxes, homo-subtypic cross-reactivity; dark grey boxes, hetero-subtypic cross-reactivity
